# microRNAs associated with the quality of follicular fluids affect oocyte and early embryonic development

**DOI:** 10.1002/rmb2.12559

**Published:** 2024-01-18

**Authors:** Sogo Aoki, Yuki Inoue, Shunsuke Hara, Jun Itou, Koumei Shirasuna, Hisataka Iwata

**Affiliations:** ^1^ Department of Animal Science, Graduate School of Agriculture Tokyo University of Agriculture Atsugi City Kanagawa Japan

**Keywords:** bovine, early embryo development, extracellular vesicle, follicular fluid, miRNA

## Abstract

**Purpose:**

Oocyte and embryo quality differs significantly among individuals. Follicular fluid (FF) is a solo environment of oocyte maturation and may flux into the oviduct. Supplementation of in vitro maturation (IVM) and culture (IVC) medium with extracellular vesicles of FFs supports oocyte maturation and embryonic development. We addressed a hypothesis that miRNA profiles in FFs are crucial background of oocyte maturation and embryonic development.

**Methods:**

FFs were collected from the ovaries of individual cows, and the FFs were classified into Good or Poor FF based on the developmental rate to the blastocyst stage of enclosed oocytes. miRNAs associated with the Good FFs were explored using small RNA sequencing. In addition, FFs were classified using the concentration of Good‐FF‐associated miRNAs. These classified FFs or miRNA were added to the IVM or IVC mediums.

**Results:**

Supplementation of IVM and IVC medium with Good FF improved embryonic development. Good FFs contained miR‐151‐3p and miR‐425‐5p at a high concentration compared with those in Poor FFs. FFs selected by the concentration of miR‐151‐3p and miR‐425‐5p improved oocyte maturation and embryonic development. Supplementation of IVM or IVC medium with either miR‐151‐3p or miR‐425‐5p improved embryonic development to the blastocyst stage.

**Conclusion:**

miRNAs were associated with the Good FFs determined oocyte maturation and embryonic development.

## Graphical abstract

miRNAs in follicular fluid support oocyte maturation and embryonic development.

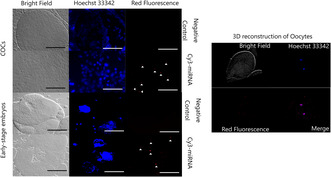

## 1 INTRODUCTION

The efficiency of blastocyst yield depends on the donor, and factors determining oocyte and embryo quality are crucial for improving embryo production. Traditionally, embryo quality has been determined by the rate of development to the blastocyst stage^1^ and morphological evaluation.^2^ The quality of oocytes is affected by the environment in which they grow^3^ and differs greatly among donors.

Follicular fluid (FF) is the sole environment surrounding cumulus cell‐oocyte complexes (COCs) in ovarian follicles. FF contains important factors that support oocyte maturation, and the addition of FF to in vitro maturation (IVM) medium improves the fertilization rate,^4^ blastulation rate,^5^ and the total cell number of blastocysts in cows.^6^ Among FF components, extracellular vesicles (EVs) have been extensively studied as key regulators of oocyte maturation. Cultured oocyte with their EVs isolated from FFs showed that the EVs were taken up by the cumulus cells,^7^ and this improved oocyte maturation^8^ along with cumulus cell expansion,^9^ and increased blastocyst yield with less blastomere apoptosis.^10^

EVs in FFs contain functional biomolecules, including lipids, proteins, RNAs, and microRNAs (miRNAs).^11^ miRNAs are single non‐coding RNA (18–25 nt) that suppress gene expression by binding to the 3′‐UTR of target mRNAs. miRNAs are thought to play a significant role in oocyte development, evidenced by the conditional knockout of DICER or Ago2, which is involved in miRNA maturation and function caused spindle formation defects and chromosomal arrangement abnormalities in oocytes.^12^, ^13^ In humans, the miRNA profiles of EVs collected from FF containing normally fertilized oocytes differ from those containing unfertilized oocytes.^14^ Human FF, which encloses oocytes with greater developmental ability, contains greater amounts of miR‐320, and the inhibition of miR‐320 in oocytes reduces blastocyst yield in mice.^15^ A previous study categorized follicles into good or poor based on the number of granulosa cell (GC)‐based miRNAs associated with good follicles. The authors also reported that supplementation with miRNAs improved in vitro growth of porcine oocytes.^16^ These reports indicate that the presence of certain miRNAs provides a background for highly competent oocytes.

FF has a wide spectrum of effects not only on oocyte maturation but also on early embryo development. Supplementation of the culture medium with FF or FF‐derived EVs changes the gene expression profiles of oviductal epithelial cells.^17^ These reports indicate that the FF flux into the oviduct regulates the cells surrounding the embryos. Consistently, the protein expression of oviductal fluids (OFs) ipsilateral to the ovulated ovary differs from those of the contralateral counterparts.^18^ Furthermore, Almiñana et al. showed that the mRNA, protein, and miRNA profiles in the EVs of OF changed before and after ovulation.^19^ We previously reported that lipofection of 8‐cell stage embryos with miRNAs detected in EV of OF around ovulation improved the blastulation rate to blastocyst,^20^ and the miRNA was found in FF at high frequencies.^21^ A report demonstrated that in vitro developing bovine embryos could take up EVs, and a greater fraction of the embryos reached the blastocyst stage with the upregulation of gene expression associated with metabolism and development,^7^ suggesting that EVs derived from FF affect early embryo development. Based on these reports, we hypothesized that miRNAs in FF determine not only the quality of oocytes but also the development of embryos, which is the underlying background of differences in donors.

Here, we rated FFs based on the developmental ability of oocytes collected from the corresponding ovaries and determined the miRNAs associated with Good FFs. We demonstrated that these miRNAs improved oocyte maturation and early embryonic development.

## 2 MATERIALS AND METHODS

### 2.1 Chemicals and medium

All chemicals were purchased from Nacalai Tesque (Kyoto, Japan) unless otherwise stated. Medium 199 (Gibco, Grand Island, NY, USA) supplemented with 5 mM taurine, antibiotics (100 IU/mL penicillin, 0.1 μg/mL streptomycin, 50 μg/mL gentamicin), and 5% fetal calf serum (FCS; 21B00A; NICHIREI BIOSCIENCES INC., Tokyo, Japan) was used for bovine granulosa cells (GCs) incubation (GC‐culture medium). Medium 199 supplemented with 10% FCS, 5 mM taurine, and antibiotics was used for in vitro maturation (IVM) of bovine oocytes. Media for in vitro fertilization (IVF) and in vitro culture (IVC) were prepared using synthetic oviductal fluid (SOF), as previously described.^20^ IVF medium consisted of SOF supplemented with 4 mg/mL fatty acid‐free bovine serum albumin and 10 IU/mL heparin. The embryos at 6 h post‐insemination were cultured in an IVC medium consisting of SOF supplemented with essential and nonessential amino acids (Sigma‐Aldrich, St. Louis, MO, USA), 1.5 mM glucose, and 1% FCS for 42 h. IVC medium supplemented with 5% FCS was used at 8‐cell‐stage embryos for 5 days. Medium 199 containing 5% exosome‐free fetal bovine serum (Exo‐FBS; System Biosciences, Palo Alto, CA, USA) instead of FCS was used for the transfection medium of GCs. The transfection medium for COCs was IVM medium containing 10% Exo‐FBS instead of FCS and that for embryos was the IVC medium containing 1% Exo‐FBS. Bovine GCs and oocytes (IVM and IVF) were incubated under atmospheric conditions of 5% CO_2_ with 95% air at 38.5°C. IVC of embryos was conducted under atmospheric conditions of 5% CO_2_ with 5% O_2_, and 90% air at 38.5°C.

### 2.2 Collection of oocytes

Ovaries were collected from Japanese Black cows at a local slaughterhouse and transferred to the laboratory within 4 h. COCs and GCs were aspirated from antral follicles (3–6 mm in diameter) using an 18 G needle (Terumo, Tokyo, Japan) connected to a 10 mL syringe (Terumo). COCs were collected from follicular contents using a stereomicroscope (Olympus, Tokyo, Japan). COCs were then subjected to IVM, IVF, and IVC protocols, as described previously.^20^ The fertilization rate and total cell number of blastocysts were examined at 18 h and 7 days post‐insemination, respectively.

### 2.3 Preparation of good and poor FF based on the blastulation rate of the corresponding oocytes

**FIGURE 1:**
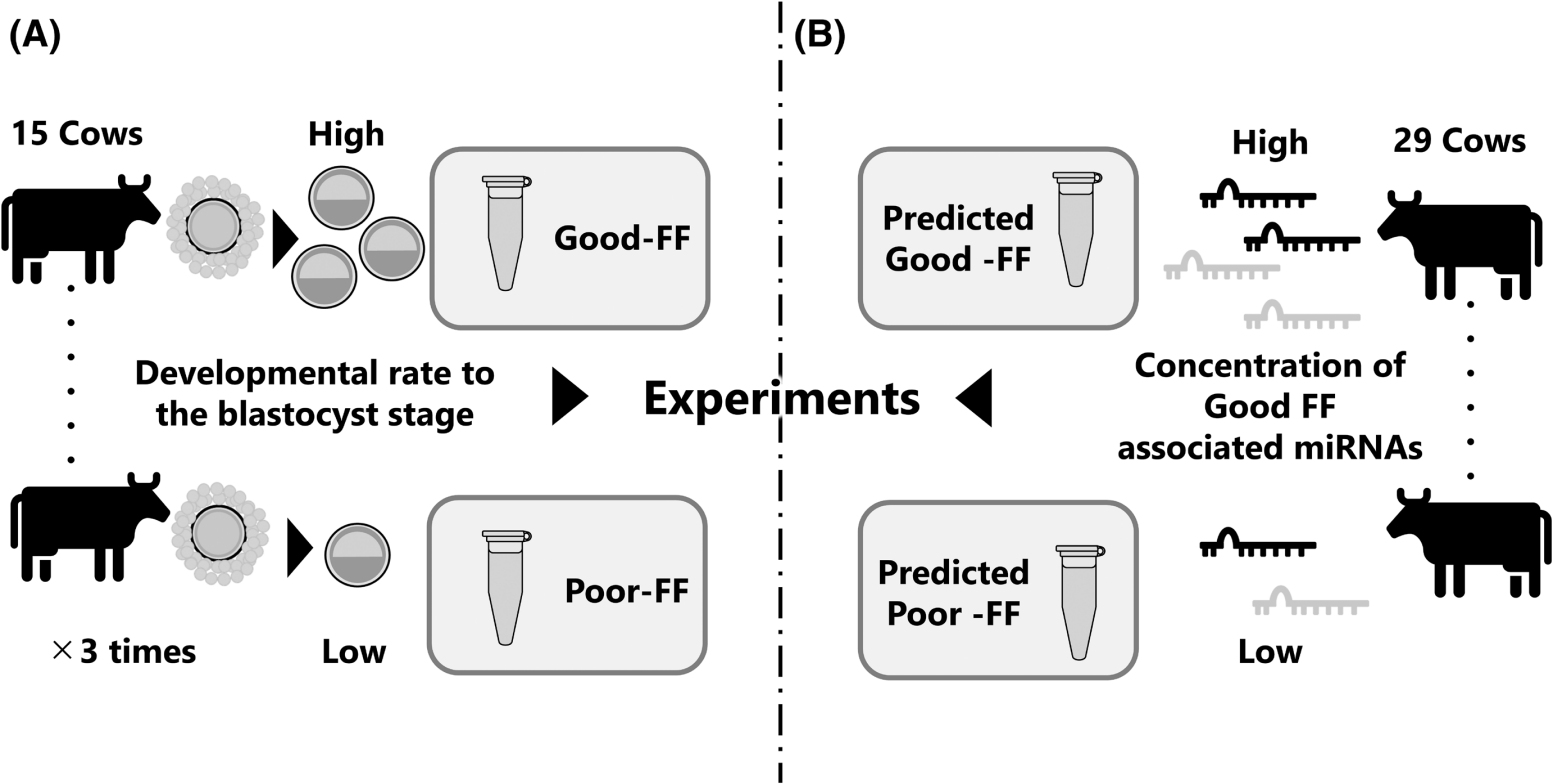
Image showing the preparation of Good and Poor FFs (A) or Predicted Good and Poor FF (B). (A) Follicular fluid (FF) was collected from the ovaries of individual cows and rated by the developmental competence of enclosed oocytes. The top 2 FFs and bottom 2 FFs were equally mixed to create Good and Poor FF. This process was repeated for 3times. (B) FFs were collected from the ovaries of individual cows and rated by the content of miR‐151‐3p and miR‐425‐5p. Top 3 and bottom 3 FFs were used for the experiments as Predicted Good FF and Poor FFs.

We collected ovaries from individual cows of an identical herd, and these corpus luteum were classified based on the previous reports.^22^ We selected 15 cows with ovaries having a mature corpus luteum (Stage III, predicted day 11–17 post‐ovulation). COCs collected individually from the ovaries of each cow were subjected to IVM, IVF, and IVC to determine the blastulation rate (blastocysts/oocytes). The remaining FFs were centrifuged at 4500 × *g* for 10 min at 25°C to remove cellular debris. The supernatants were filtered through a 0.20‐μm pore filter (Minisart, Sartorius Stedim Biotech GmbH, Göttingen, Germany) to remove vesicles larger than 200 nm and stored at −80°C until use. Based on the blastulation rate of the corresponding oocytes, Good FF (top two cows) and Poor FF (bottom two cows) groups were selected. Good and Poor FFs were equally mixed and used for the experiments. This process was repeated thrice to produce three batches of Good FF and Poor FF (Figure 1A).

### 2.4 Effect of supplementation of IVM medium with good or poor FFs on oocyte development

COCs were cultured in an IVM medium supplemented with 10% Good FF or Poor FF for 21 h and subjected to IVF. The concentration of FFs was determined by the previous report.^23^ After 48 h post‐insemination, embryos were examined for cleavage rate to 8‐cell stage embryos and subsequently incubated in IVC medium containing 5% FCS for 5 days, and the blastulation rate and total cell number of the blastocysts were examined.

### 2.5 Examination of the fertilization rate and total cell number of blastocysts

Eighteen hours after post‐insemination, the presumptive zygotes were soaked in acetic ethanol (1:3) for 15 min, and the presence of pronuclei was evaluated using a stereomicroscope (Olympus). Zygotes with two pronuclei were considered to have normal fertilization, and the others were considered abnormal. For counting the total cell number of blastocysts, embryos were fixed in 4% paraformaldehyde, stained with Hoechst 33342 (1 μg/mL) in PBS, and observed under a fluorescence microscope (DMI 6000 B; Leica, Wetzlar, Germany).

### 2.6 Effect of supplementation of the IVC medium with good or poor FFs on the embryonic development

Eighteen hours after insemination, presumptive zygotes were incubated in the IVC medium containing 1% Good FF or 1% Poor FF for 30 h. The concentration of FFs was determined by preliminary experiments (Figure S1) and a presumption that concentration of FFs remained in the oviduct was low. The cleavage rate of the 8‐cell stage embryos was examined, and the embryos were subsequently incubated in an IVC medium for 5 days; subsequently, the blastulation rate and total cell number of the blastocysts were examined as described above.

### 2.7 EV isolation and RNA extraction from FF‐EV

EVs were isolated from FFs using ExoQuick (EXOQ20A‐1, System Biosciences) following the manufacturer's instructions. Briefly, before extraction, collected FFs were centrifuged at 4500 × *g* for 10 min (greater than that of the setting in the protocol: 3000 × *g*) to remove cellular debris followed by filtration through a 0.20‐μm pore filter (Minisart, Sartorius Stedim Biotech GmbH) to remove vesicles larger than 200 nm. 500 μL Good and Poor FFs were mixed with ExoQuick and incubated overnight at 4°C followed by centrifugation (15 000 × *g* for 2 min) to obtain a corresponding pellet of Evs. miRNAs in Evs were extracted using the SeraMir Exosome RNA Amplification Kit (System Biosciences, RA806A‐1) and used for small RNA‐seq. Sohel et al. showed that Evs isolated from bovine FFs using this kit were similar to their counterparts obtained after ultracentrifugation in size and protein levels of CD63, known to be expressed in the membrane of Evs.^24^ Furthermore, they showed that the extracted Evs did not contain mitochondrial protein indicating that the EV is free of any protein of cellular origins. From these, the Evs meet MISEV guideline.

### 2.8 Small RNA‐sequences of miRNAs

The quality and concentration of RNA were examined using a Bioanalyzer (Agilent Technologies, Palo Alto, CA, USA), and a cDNA library of RNAs from Evs extracted from FFs was constructed using the NEBNext Multiplex Small RNA Library kit (New England Biolabs, Ipswich, MA, USA). The average length of all the derived libraries was confirmed using an Agilent Bioanalyzer with a High Sensitivity DNA Kit (Agilent Technologies, Palo Alto, CA, USA), and the concentration of each library was adjusted to 10 nM based on the qPCR results (KAPA Biosystems, Boston, MA, USA). Multiplexed samples were sequenced as 75 single‐read cycles on a NextSeq 500 system (Illumina). Image analysis, base calling, and quality filtering were performed using Real‐Time Analysis version 2.4.11 (Illumina). After sequencing, the data were processed in Fastq using the bcl2fastq‐v2.20.0.422. Raw data were confirmed to be of high quality after discarding adapter sequences, ambiguous nucleotides, and low‐quality sequences. The data were filtered to identify sequences with lengths of mature miRNAs ranging from 18 to 25 nt and mapped against sequences in the miRbase v22 (https://www.mirbase.org/) using CLC‐quantifying miRNA tools to obtain the total count sequence reads. The sequence data of miRNAs in Evs extracted from FFs were registered in the DDBJ Read Archive under the accession number DRA016921.

### 2.9 Collection and preparation of live GCs

Follicular contents were aspirated from the antral follicles as described above. After the collection of COCs, the remaining follicular contents were filtered through a 75 μm cell strainer to remove cellular debris, followed by centrifugation at 200 × *g* for 1 min at 25°C to obtain a cellular pellet. The GCs were incubated overnight in Medium 199 containing 5% FCS in a culture plate (90 mm; Sarstedt Inc., Sarstedt, Germany) overnight. Live GCs attached to the culture plates were collected using Accumax (Innovative Cell Technologies) and used for the transfection of miRNA or EV uptake assays.

### 2.10 Preparation of the pmirGLO vector and miRNA mimics

**FIGURE 2:**
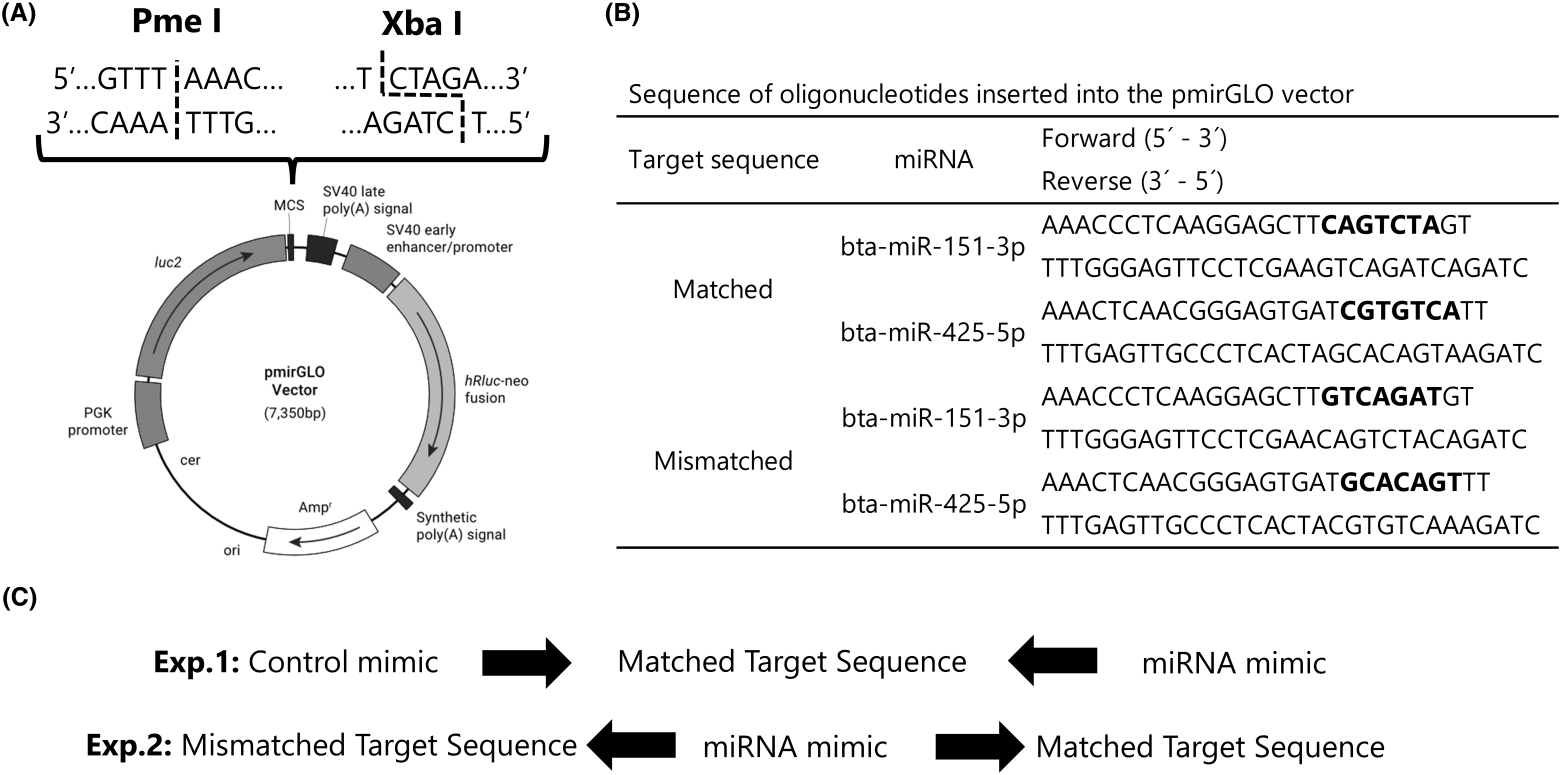
Schematic design of the pmirGLO vector and experimental design. (A) pmirGLO Dual‐Luciferase miRNA Target Expression Vector and (B) matched or mismatched sequence of miRNAs (miR‐151‐3p and miR‐425‐5p). (C) Two combinations of pmirGLO vector and miRNA mimics for dual‐luciferase assay. Exp. 1: The recombinant vector having matched sequences of miRNAs and miRNAs or control mimics was co‐transfected into GCs. Exp. 2: The recombinant vector having matched or mismatched sequence and miRNA mimics was co‐transfected into GCs.

Oligonucleotides, including matched and mismatched target sequences of each miRNA (miR‐151‐3p and miR‐425‐5p), are shown in Figure 2A,B. These sequences were inserted downstream of the firefly luciferase gene in the pmirGLO Dual‐Luciferase miRNA Target Expression Vector (Promega, Madison, WI, USA) between the PmeI/XbaI sites. miRNA control mimics, miR‐151‐3p and miR‐425‐5p mimics were purchased from miRVana (Ambion, Grand Island, NY, USA; miRNA mimic Negative control #1: cat #4464059; bta‐miR‐151‐3p: cat #4464066, 5′‐ CUAGACUGAAGCUCCUUGAGG‐3′; bta‐miR‐425‐5p: cat #4464066, 5′‐ AUGACACGAUCACUCCCGUUGA‐3′).

### 2.11 Dual‐luciferase reporter assay

For the dual‐luciferase assay, live GCs (see Section 2.9) were incubated overnight in a 96‐well dish (BD Biosciences, Franklin Lakes, NJ, USA) at a density of 2.0 × 10^4^ cells/well. Then, the cellular viability and concentration (70% confluence) were checked under a microscope and used for transfection using Lipofectamine®3000 (Invitrogen, Carlsbad, CA, USA) and a selective combination of miRNA mimic (final concentration of 60 nM) and pmirGLO vector in the transfection medium of GCs (Section 2.1). Transfection of miRNA mimics was validated using two settings: a selective combination of miRNA mimics and the pmirGLO vector (Figure 2C, Exp.1 and 2). Two days after transfection, the luciferase activity of the cells was analyzed using the Dual‐Glo® Luciferase Assay System (Promega) and a luminometer (Spark 10 M; Tecan Japan Co., Ltd., Kanagawa, Japan) following the manufacturer's instructions. The expression levels of firefly luciferase were normalized to the activity of the Renilla luciferase.

### 2.12 Transfection of GCs with Cy3‐labeled miRNA mimics and isolation of EVs secreted from GCs

The miRNA control mimic was labeled with cyanine dye 3 (Cy3) using a Label IT siRNA Tracker Cy3 Kit (Mirus, Madison, WI, USA) following the manufacturer's instructions. Live GCs (section 2.9.) were cultured overnight in a 4‐well plate at a density of 2.0 × 10^5^ cells per well and transfected with Cy3‐labeled miRNA control mimic (Cy3‐miRNA, 60 nM) using Lipofectamine®2000 (Invitrogen) and transfection medium. One day after lipofection, the GCs were washed, and the medium was replaced with a fresh transfection medium. After 24 h of incubation, the medium was collected, and 5 mL was centrifuged (700 × *g* for 5 min) followed by filtration through a 0.2‐μm pore filter (Minisart). Evs in the medium were isolated using ExoQuick‐TC (EXOTC10A‐1; System Biosciences) and diluted in PBS (500 μL). The Evs were used for EV uptake assay in COCs or embryos at a concentration of 1%.

### 2.13 GC and embryos EV uptake assay

COCs were co‐incubated with Evs containing Cy3‐miRNA as described in Section 2.12 for 21 h. Then, the COCs or denuded oocytes were fixed in 4% paraformaldehyde overnight. Presumptive zygote (18 h post‐insemination) were co‐incubated with Evs containing Cy3‐miRNA for 30 h. After incubation, 8‐cell‐stage embryos were collected and fixed in 4% paraformaldehyde overnight. Before fixation, the zona pellucida (ZP) of the embryos was removed using 0.5% proteinase (Sigma Aldrich) to obtain clear Cy3 signals. The fixed COCs, oocytes, and embryos were stained with Hoechst 33342 (1 μg/mL) in PBS and mounted on glass slides using Immunoselect Antifading Mounting Medium (SCR‐038447; Dianova, Hamburg, Germany). Cumulus cells, oocytes, and embryos were examined by fluorescence microscope (DMI 6000 B; Leica). Z‐stack fluorescent images of the samples were acquired to detectCy3 signals in cells, oocytes, and embryos.

### 2.14 Effect of supplementation of the IVM medium with miRNA mimics on the embryonic development

COCs were transfection using Lipofectamine® 2000 (Invitrogen) and miRNA mimics (control, miR‐151‐3p, or miR‐425‐5p) by incubating in the transfection medium of oocytes (Section 2.1) for 21 h. The miRNA mimic concentration was set at 60 nM following pre‐experiment where transfection efficiency was examined using the BLOCK‐iT™ Alexa Fluor™ Red Fluorescent Control (14750‐100, Invitrogen). After 21 h of incubation, the COCs were subjected to IVF and IVC. Culture experiments were repeated 18 times.

### 2.15 Effect of supplementation of IVC medium with miRNA mimics on the embryonic development

ZP blocks the lipofection of siRNAs.^25^ Therefore, 18 h after insemination, presumptive zygotes were treated with 0.5% proteinase (Sigma Aldrich) to remove the ZP, and then, the embryos were subjected to lipofection following a previously reported protocol.^20^ Embryos were transfected using Lipofectamine® 2000 (Invitrogen) with miRNA mimics (control, miR‐151‐3p, or miR‐425‐5p) in the transfection medium (Section 2.1) for 30 h. The embryos were washed and incubated in the IVC medium for 5 days. Culture experiments were repeated 20 times.

### 2.16 Selection of FFs based on miRNAs content

To validate the relationship between Good FF and miR‐151‐3p and miR‐425‐5p, FFs were rated based on the concentration of miRNAs. 29 cows having ovaries (Grade 3) were selected, miRNAs were extracted from the FF of 29 cows, and the miRNA quantity was examined using Mir‐X miRNA RT‐qPCR TB Green kit (Takara Bio, Siga, Japan) according to the manufacture‐provided methods (Figure 1B). Primers used for the detection of miR‐151‐3p and miR‐425‐3p were sequences of the corresponding miRNAs obtained from the miRbase. The concentration of miRNAs was normalized using the expression value of miR‐103.^24^ Based on the results, top 3 and bottom 3 FFs were selected. These FFs were used for supplementation of IVM or IVC medium as predicted Good or Poor FFs.

### 2.17 Enriched pathways related to the in silico‐predicted target genes

In silico prediction of the target genes of miRNAs (miR‐151‐3p and miR‐425‐5p) was conducted using TargetScan (http://www.targetscan.org), with “cow” selected as the background and species. Enriched pathways based on the Kyoto Encyclopedia of Genes and Genomes (KEGG) related to the target genes were predicted using the Database for Annotation, Visualization, and Integrated Discovery (DAVID, https://david.ncifcrf.gov/) functional annotation tool, with *Bos taurus* as the background and species.

### 2.18 Statistical analyses

Data are presented as mean ± standard error of the mean (SEM). Data were analyzed using the Shapiro–Wilk test to determine normality. For comparison between the two groups, we used Student's *t*‐test for data with normal distribution and the Mann–Whitney *U*‐test for data with non‐normal distribution. The Kruskal–Wallis test followed by the Steel‐Dwass test was used for comparison among the three groups. Statistical significance was set at *p* < 0.05.

## 3 RESULTS

### 3.1 FFs rated based on enclosed oocyte developmental ability improved fertilization rate and early embryonic ability

Supplementation of IVM medium with Good FF improved the normal fertilization rate compared to Poor FF (Table 1). Good FF significantly improved cleavage and blastulation rates (Table 1). However, the total cell numbers of the blastocyst were comparable between the two groups (Table 1). Supplementation of the IVC medium with Good FF significantly improved the cleavage rate, blastulation rate, and total cell number of blastocysts compared to their Poor FF counterparts (Table 2).

**TABLE 1:** Rate of fertilization, cleavage, and blastulation of embryos derived from Good or Poor FF‐treated oocytes.

FFs (10%)	Trial	Oocytes	Normal (%)	Abnormal (%)	Trial	Oocytes	Cleavage (%)	Blastulation (%)	Total cell number
Good	6	84	82.1 ± 2.3**a**	17.9 ± 2.3**a**	21	257	52.5 ± 1.8**a**	19.4 ± 1.1 **a**	128.6 ± 5.3 (32)
Poor	6	81	66.7 ± 4.2**b**	33.3 ± 4.2**b**	21	257	43.0 ± 1.2**b**	14.6 ± 0.8 **b**	119.7 ± 5.6 (25)

*Note*: Good and Poor FF were added to the IVM medium. The fertilization rate at 18 h post‐insemination, cleavage rate at 48 h post‐insemination, and blastulation rate 7 days post‐insemination were examined. Trial: number of trial, Oocytes: number of oocytes, Normal: normal fertilization rate, Abnormal: abnormal fertilization rate, Cleavage: cleavage rate, Blastulation: blastulation rate to blastocyst. Data are presented as mean ± SEM. a, b; *p* < 0.05.

**TABLE 2:** Effect of FF treatment to IVC medium on rate of cleavage and blastulation of embryos.

FFs (1%)	Trial	Zygotes	Cleavage rate (%)	Blastulation rate (%)	Total cell number
Good	15	179	64.8 ± 4.2a	26.8 ± 2.5a	154.8 ± 8.5 (38)a
Poor	15	179	48.5 ± 2.8b	13.9 ± 1.9b	116.2 ± 6.6 (20)b

*Note*: Presumptive zygotes (18 h post‐insemination) were incubated with 1% Good FF or Poor FF for 30 h. Cleavage rate (>8‐cell stage) at 48 h post‐insemination, and blastulation rate 7 days after insemination were determined. Trial: number of trial, Zygotes: number of zygotes. Data are presented as mean ± SEM. a, b; *p* < 0.05.

### 3.2 miRNA profiles for good and poor FF

Small RNA‐seq detected 561 and 577 miRNAs in EVs of Good FF and Poor FF groups, respectively. The top 10 high‐frequency miRNAs are listed in Table 3, and almost all miRNAs overlapped and did not differ significantly between the two groups (Table 3). Seven miRNAs were expressed at significantly higher levels in the Good FF compared to the Poor FF (Table 4). The top two miRNAs, miR‐151‐3p and miR‐425‐5p, were selected and used for subsequent experiments.

**TABLE 3:** Top 10 miRNAs in EVs extracted from FFs with high frequency.

Good FF		Poor FF	
miRNA	Read count	miRNA	Read count
bta‐miR‐148a	130,029.7	bta‐miR‐148a	125,989.4
bta‐let‐7i	58,465.3	bta‐miR‐30d	44,540.6
bta‐miR‐30d	46,826.7	bta‐let‐7i	42,956.2
bta‐miR‐26a	41,145.0	bta‐miR‐92a	40,374.0
bta‐miR‐92a	40,141.3	bta‐miR‐26a	37,853.8
bta‐let‐7 g	35,265.3	bta‐miR‐25	31,833.8
bta‐miR‐25	26,989.7	bta‐let‐7 g	30,481.4
bta‐miR‐21‐5p	23,768.7	bta‐miR‐21‐5p	29,827.6
bta‐miR‐378	19,087.3	bta‐miR‐10b	25,378.8
bta‐miR‐30a‐5p	18,855.0	bta‐miR‐30a‐5p	24,253.4

*Note*: Top 10 abundant miRNAs found in Good or Poor FFs identified from small RNA‐seq. Data show the average value of read count.

**TABLE 4:** Significantly expressed miRNAs in EVs of Good FF.

miRNAs	Read count	*p*‐Value
miR‐151‐3p	335.0	0.0486
miR‐425‐5p	259.3	0.0336
miR‐6517	63.7	0.0254
miR‐11 976	18.0	0.0004
miR‐423‐5p	8.3	0.0150
miR‐21‐3p	5.7	0.0465
miR‐11 972	3.7	0.0391

*Note*: miRNAs presented in Good FF with higher frequency compared to those in Poor FF. Data show the average value of read count.

### 3.3 miR‐151‐3p and miR‐425‐5p mimics function in bovine GCs

**FIGURE 3:**
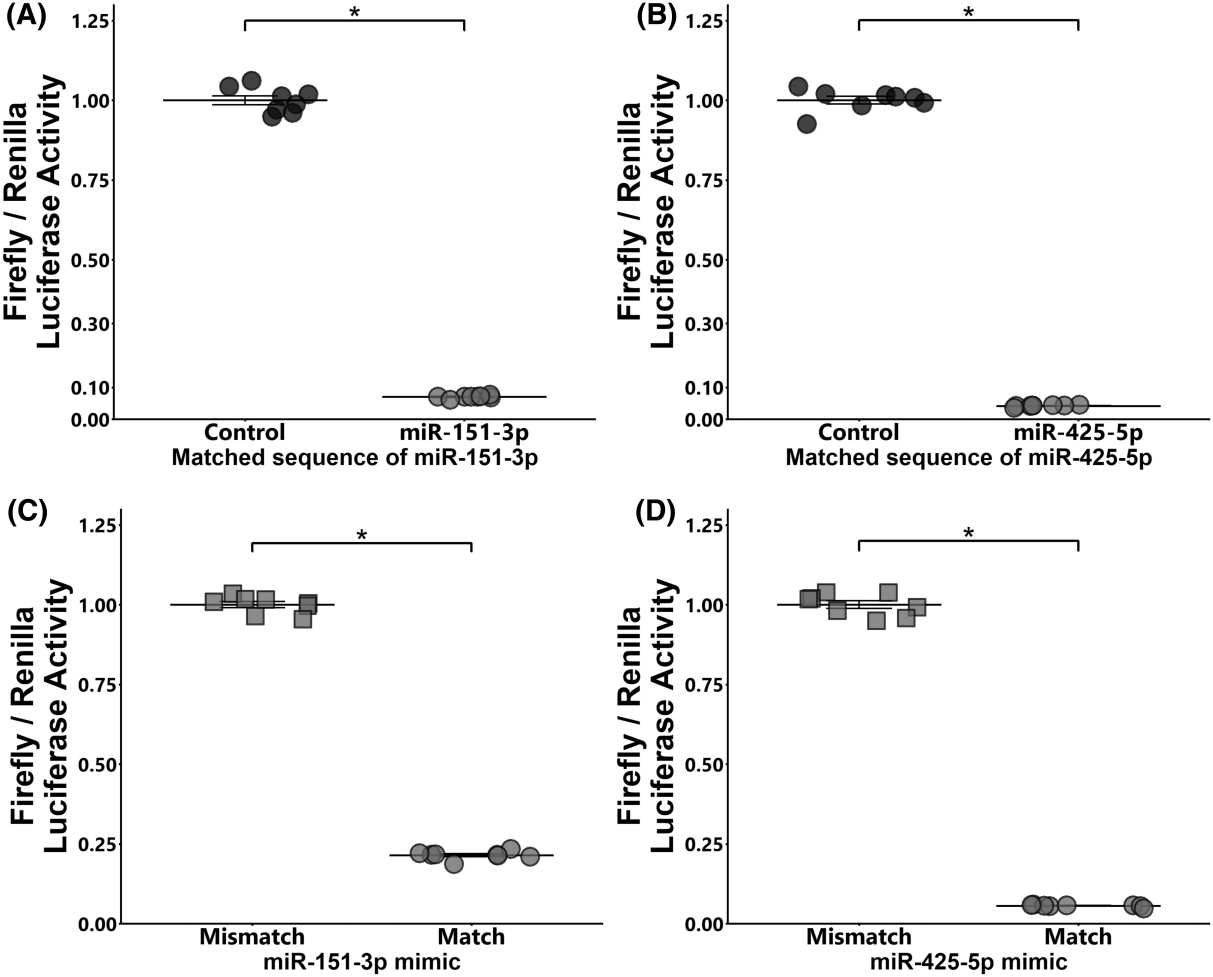
Dual‐luciferase reporter assay. (A, B) GCs were transfected with pmirGLO‐vector with the matched target sequence of miRNAs and then co‐transfected with the miRNAs (A: miR‐151‐3p, B: miR‐425‐5p) or control mimic. The expression value of the control is defined as 1.0. (C, D) GCs were transfected with pmirGLO‐vector with matched or mismatched target sequences and then co‐transfected with miRNA mimics (C: miR‐151‐3p, D: miR‐425‐5p). The average value of mismatch was defined as 1.0. Each experiment consists of 8 replicates. Data are presented as mean ± SEM, *p < 0.05.

To confirm whether miRNA mimics function in bovine cells, a dual‐luciferase reporter assay was conducted. miR‐151‐3p and miR‐425‐5p mimics reduced the ratio of Firefly/Renilla luciferase expression in GCs transfected with the pmirGLO vector having a matched sequence compared to that of control mimic counterparts (miR‐151‐3p mimic: 0.07‐fold, *p < 0.05; miR‐425‐5p mimic: 0.04‐fold, *p < 0.05, Figure 3A,B). Both mimics significantly reduced the ratio of Firefly/Renilla luciferase expression in GCs transfected with the pmiR‐GLO vector with a matched targeting sequence compared to their mismatched counterparts (miR‐151‐3p: 0.21‐fold; miR‐425‐5p: 0.06‐fold, *p < 0.05, Figure 3C,D).

### 3.4 Cumulus cells, oocytes, and embryos uptake the miRNA in EVs released from GCs

**FIGURE 4:**
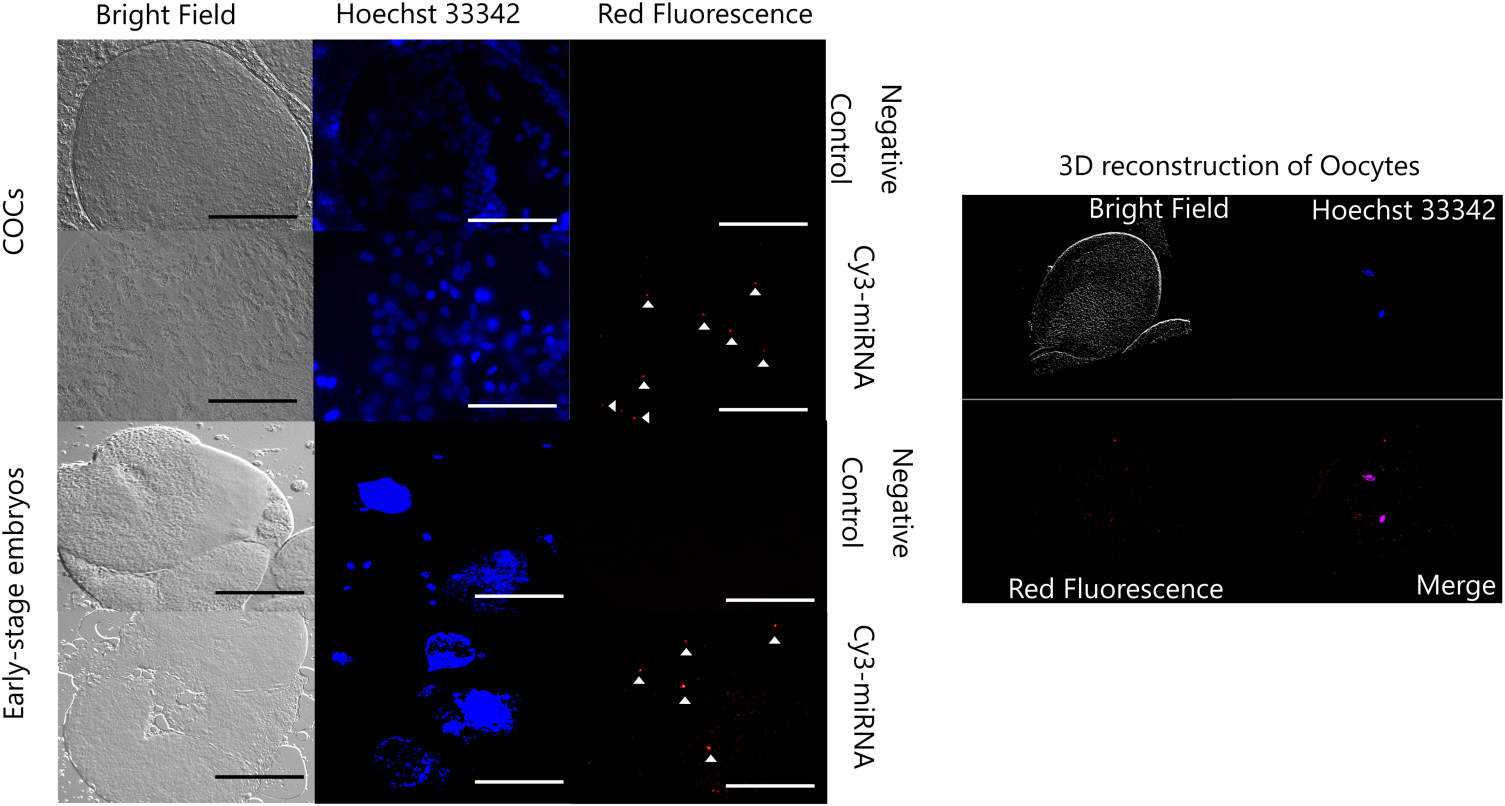
Representative images of COCs, denuded oocytes, and blastomere of 8‐cell stage embryos treated with EV‐enclosed Cy3‐miRNA. EVs were co‐incubated with COCs or embryos for 24 or 30 h, respectively. Vehicle‐treated GCs and embryos (treated with PBS) are negative controls. The image of oocytes was reconstructed using z‐stack function and Leica LAS AF software. The white triangle indicates the positive signals for red fluorescence. Scale bar indicates 100 μm.

After co‐incubation of EVs enclosed Cy3‐miRNA with COCs or embryos, positive signals (Cy3) were detected in cumulus cells, oocytes, and blastomeres (Figure 4).

### 3.5 miR‐151‐3p and miR‐425‐5p mimics significantly improve embryonic developmental ability

**FIGURE 5:**
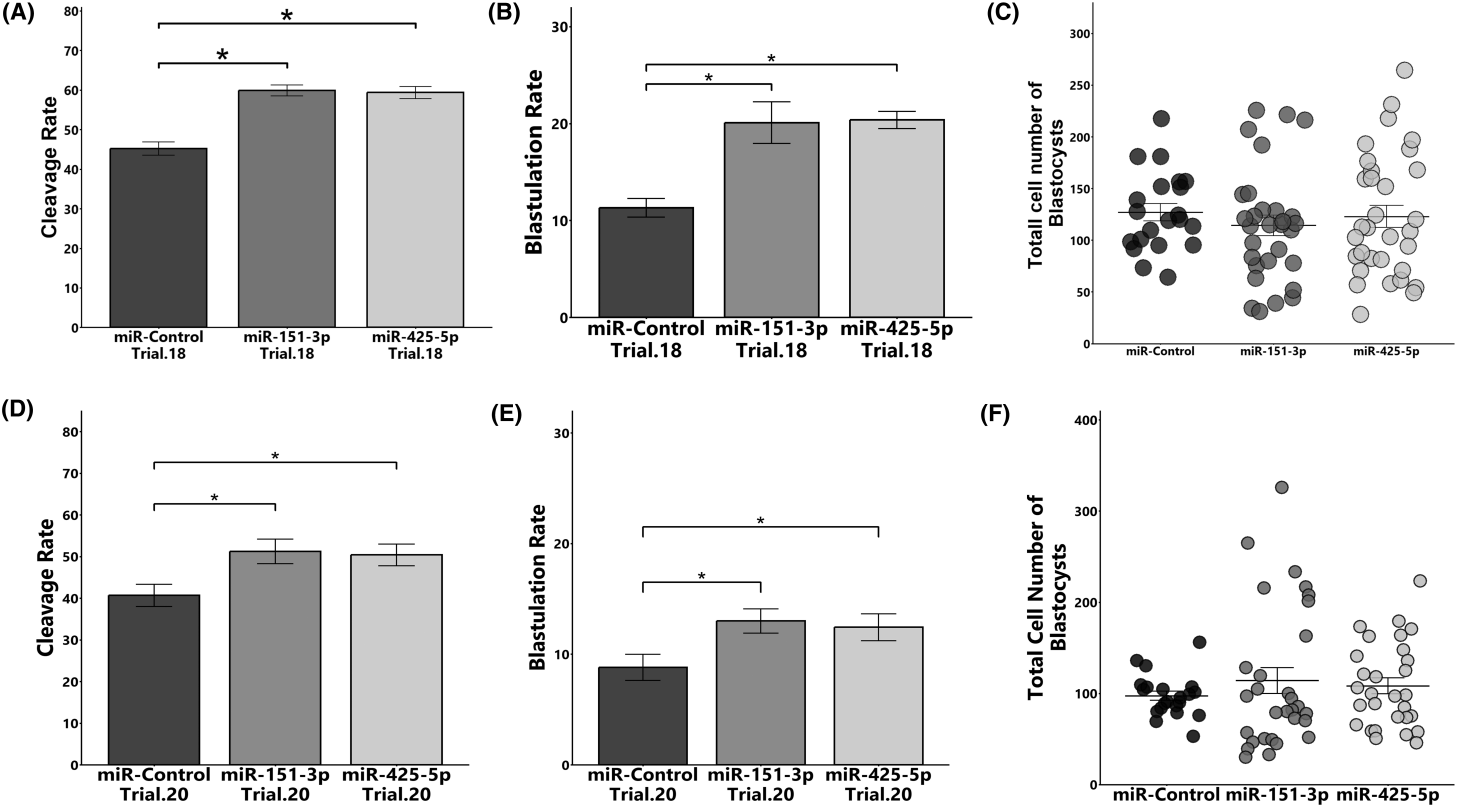
Effect of miR‐151‐3p and miR‐425‐5p on the cleavage rate, blastulation rate, and total cell number of blastocysts. (A–C) miRNA mimics were added to the IVM medium and the cleavage rate (A), blastulation rate (B), and total cell number of blastocysts (C) were determined. D‐F: Zona pellucida‐free presumptive embryos (18 h post‐insemination) were cultured with miRNA or control mimics for 30 h, and the cleavage rate (D), blastulation rate (E), and total cell number of blastocysts (F) were determined. Total number of oocyte and blastocysts used for each group were as follows, (A, B) Control: *n* = 188, miR‐151‐3p: *n* = 188, miR‐425‐5p: *n* = 188, (C) Control: *n* = 21, miR‐151‐3p: *n* = 30, miR‐425‐5p: *n* = 32, (D, E) Control: *n* = 240, miR‐151‐3p: *n* = 240, miR‐425‐5p: *n* = 240, (F) Control: *n* = 21, miR‐151‐3p: *n* = 30, miR‐425‐5p: *n* = 29. Data are presented as mean ± SEM, *p < 0.05.

The transfection of COCs with miR‐151‐3p and miR‐425‐5p mimics during IVM significantly improved the cleavage rate (control: 45.2 ± 1.7% vs. miR‐151‐3p: 59.9 ± 1.4% vs. miR‐425‐5p: 59.4 ± 1.5%, *p < 0.05, Figure 5A) and the blastulation rate (control: 11.3 ± 0.93% vs. miR‐151‐3p: 20.1 ± 2.1% vs. miR‐425‐5p: 20.4 ± 0.86%, *p < 0.05, Figure 5B) compared with those of control mimics.

Similarly, transfection of presumptive zygotes (from zygotes to 8‐cell‐stage) with both miRNA mimics significantly improved the cleavage rate (control: 40.7 ± 2.6% vs. miR‐151‐3p: 51.2 ± 2.9% vs. miR‐425‐5p: 50.4 ± 2.5%, *p < 0.05, Figure 5D) and the blastulation rate (control: 8.8 ± 1.2% vs. miR‐151‐3p: 13.0 ± 1.1% vs. miR‐425‐5p: 12.4 ± 1.2%, *p < 0.05, Figure 5E) compared with those of control mimics. However, the total cell number of the blastocysts did not differ among groups (Figure 5C,F).

### 3.6 Effect of predicted good and poor FFs on oocyte maturation and embryonic development

**FIGURE 6:**
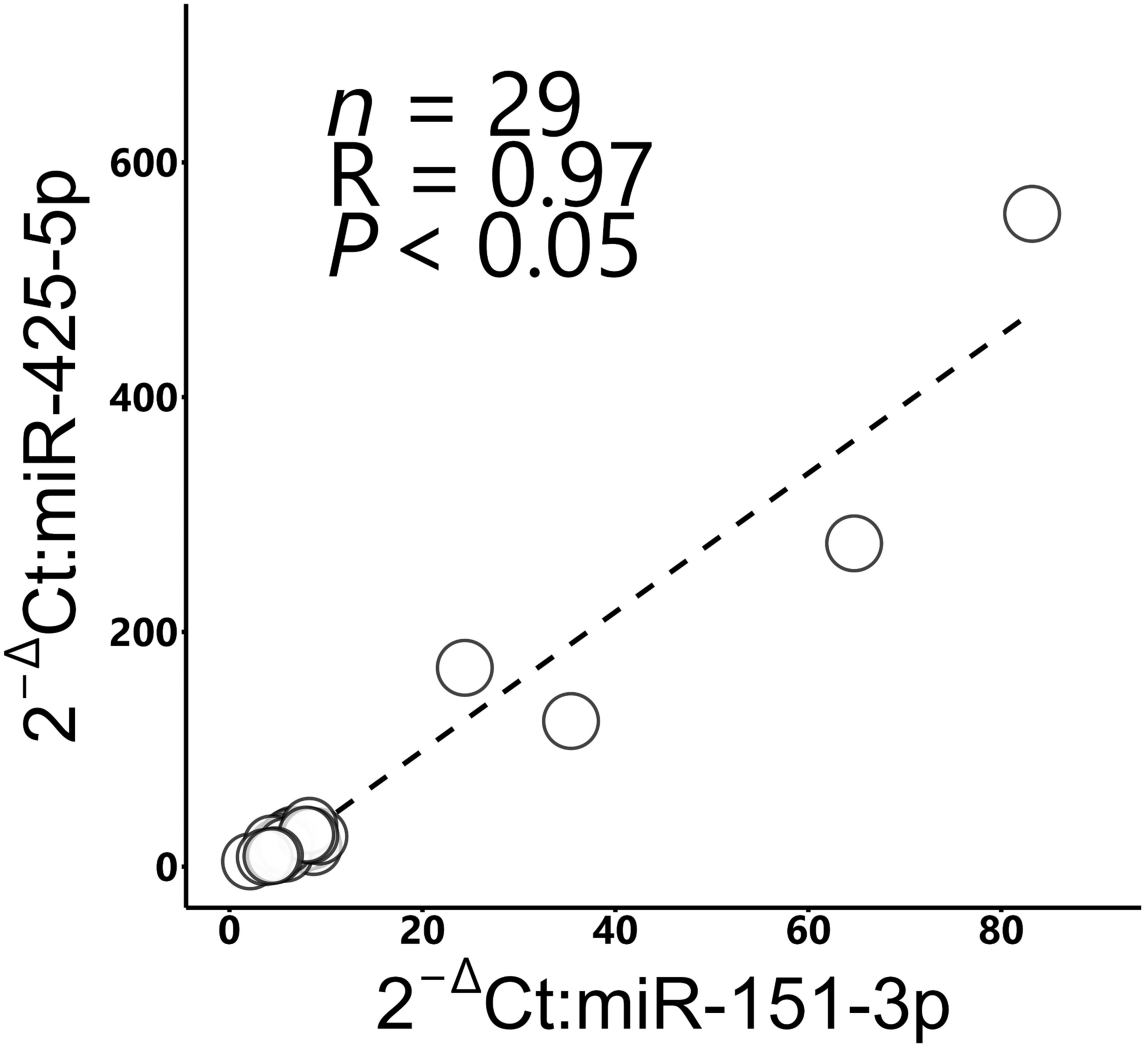
The correlation between the content of miR‐151‐3p and miR‐425‐5p in FFs of 29 cows.

**FIGURE 7:**
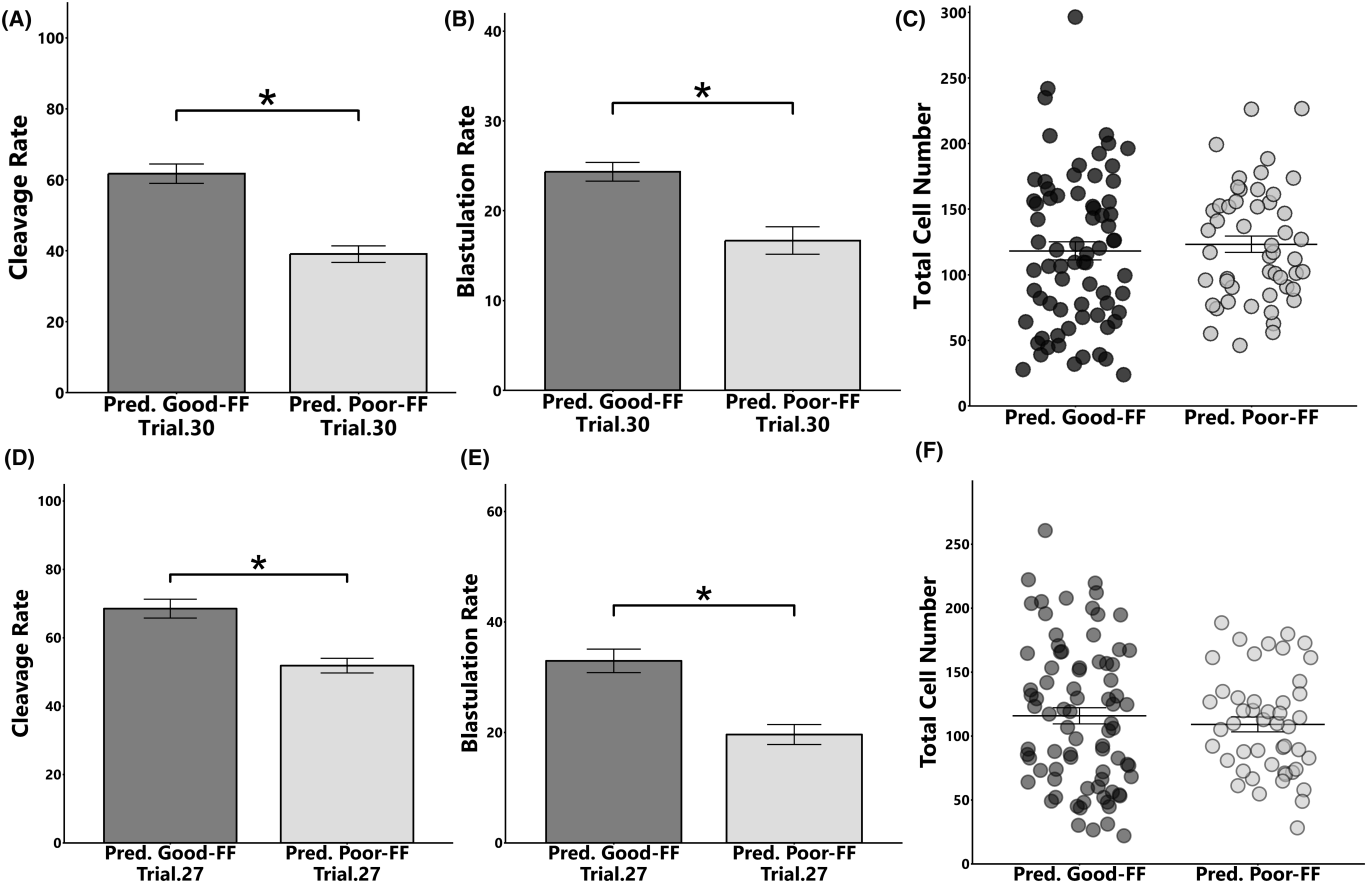
Effect of Predicted Good FF or Predicted Poor FF on the cleavage rate, blastulation rate, and total cell number of blastocysts. (A–C) Predicted FFs were added to the IVM medium, and the cleavage rate (A), blastulation rate (B), and total cell number of blastocysts (C) were determined. D‐F: Presumptive zygotes (18 h post‐insemination) were cultured with Predicted Good FFs or Poor FF for 30 h, and the cleavage rate (D), blastulation rate (E), and total cell number of blastocysts (F) were determined. Total number of oocyte and blastocysts used for each group were as follows, (A, B) Predicted Good FF: *n* = 300, Predicted Poor FF: *n* = 300, (C) Predicted Good FF: *n* = 72, Predicted Poor FF: *n* = 50, (D, E) Predicted Good FF: *n* = 270, Predicted Poor FF: *n* = 270, (F) Predicted Good FF: *n* = 80, Predicted Poor FF: *n* = 47. Data are presented as mean ± SEM, *p < 0.05.

Reverse transcript PCR showed a significant positive correlation between the expression levels of miR‐151‐3p and miR‐425‐5p in FFs (*r* = 0.97, *p* < 0.05, Figure 6). In addition, supplementation of IVM and IVC medium with the predicted Good FF significantly improved the cleavage rate (IVM: 61.7 ± 2.7% vs. 39.0 ± 2.3%, *p < 0.05, Figure 7A; IVC: 68.5 ± 2.7% vs. 51.9 ± 2.1%, *p < 0.05, Figure 7D) and blastulation rates (IVM: 24.3 ± 1.0% vs. 16.7 ± 1.5%, *p < 0.05, Figure 7B; IVC: 33.0 ± 2.1% vs. 19.6 ± 1.8%, *p < 0.05, Figure 7E) compared to those of predicted Poor FF. However, the total cell number of the blastocysts did not differ among groups (Figure 7C,F).

### 3.7 Enriched KEGG pathways related to the target genes of miR‐151‐3p and miR‐425‐5p

In silico prediction of 96 (miR‐151‐3p) and 1622 (miR‐425‐5p) target genes revealed significantly enriched 27 and 82 pathways for miR‐151‐3p and miR‐425‐5p, respectively (*p* < 0.05, Table S1). The top five pathways for each miRNA are listed in Table 5.

**TABLE 5:** Top five KEGG pathways related to the target genes of miRNAs.

Terms	Count	*p*‐Value
KEGG Pathway enriched by target genes of miR‐151‐3p		
Rap1 signaling pathway	5	4.34.E−03
cAMP signaling pathway	5	6.74.E−03
MAPK signaling pathway	5	1.47.E−02
Alzheimer disease	5	4.90.E−02
HIF‐1 signaling pathway	4	2.14.E−03
KEGG Pathway enriched by target genes of miR‐425‐5p		
Human papillomavirus infection	37	5.25E−03
MAPK signaling pathway	36	3.53E−04
PI3K‐Akt signaling pathway	36	2.56E−02
Proteoglycans in cancer	32	6.92E−06
Calcium signaling pathway	32	4.41E−04

*Note*: KEGG pathways enriched by in silico predicted target genes (Target Scan) for miR‐151‐3p or miR‐425‐5p. Pathways were predicted using the DAVID functional annotation tool.

## 4 DISCUSSION

The present study showed that the supplementation of IVM or IVC medium with Good FF significantly improved oocyte and early embryo development to the blastocyst stage. miRNAs are key factors underlying the beneficial effects of FF.

The beneficial effects of supplementing FFs to the IVM medium on oocyte maturation and subsequent embryonic development have been reported in humans,^26^ dogs,^27^ horses,^28^ pigs,^29^ and cows.^4^, ^5^, ^6^, ^23^, ^30^ The developmental ability of the enclosed oocytes was linked to the effect of FFs on IVM, indicating that FF, which originates is from middle antral follicles, differs among donors and is a causal factor for differences in oocyte quality at the individual levels.

Previous reports have indicated that EVs in FFs are crucial factors underlying mutual interactions between FFs and GCs.^11^ da Silveira et al. showed that cumulus cells surrounding oocytes take up signal‐conjugated EV.^7^ The addition of FF‐EVs to the IVM medium improves oocyte developmental competence in cows.^7^, ^31^ Consistently, miRNA in EVs derived from GCs were taken up by cumulus cells, oocytes, and embryos indicating the direct effect of miRNAs contained in the EVs on these cells. miRNAs in EVs of FF are pivotal for oocyte growth because supplementation of the culture medium with miRNAs improves oocyte growth in cows and pigs.^16^, ^21^ Based on these reports, we speculated that miRNA profiles might differ between Good and Poor FF. Small RNA‐seq of the FF‐EV showed that the top 10 miRNAs in EVs both Good and Poor FF groups overlapped. The miRNA profiles of the FFs found in the present study also overlapped with those reported in cows.^21^, ^24^ Furthermore, many miRNAs found in human FFs overlap with the miRNAs found in this study.^32^ Based on these reports, the top miRNAs in the EVs of FF are conserved among cows and resemble those in humans. Seven miRNAs with significantly higher expression in the Good FF than in Poor FF groups were found and the top two miRNAs (miR‐151‐3p and miR‐425‐5p) were used for the experiment. Moreover, these miRNAs were found in FFs of bovine preovulatory follicles (DRA016922) indicating that miR‐151‐3p and miR‐425‐5p are present in the FF during oocyte maturation. The present study demonstrates that supplementation of IVM medium with the miRNAs improved oocyte developmental competence. Consistently, Sørensen et al. showed low expression levels of miR‐151‐3p in the FFs of patients with polycystic ovary syndrome (PCOS),^33^ suggesting that miR‐151‐3p is a marker of healthy ovarian conditions. Hu et al. showed higher miR‐425‐5p expression in medium‐sized follicles (3–5 mm) than that of small follicles (<3 mm) in pigs,^34^ suggesting that miR‐425‐5p may be associated with follicle development. Furthermore, FFs containing high miR‐151‐3p and miR‐425‐5p (Predicted Good FF) improved oocyte maturation suggesting that miR‐151‐3p and miR‐425‐5p are potential markers for Good FF and good‐quality oocytes. Notably, the present study demonstrates that miRNA treatment of cumulus cells is beneficial for oocyte competence, since miRNA mimic was added to COCs in which ZP of oocytes was intact. However, the findings also indicate that miRNAs contained in EVs can enter oocytes. Therefore, transfection assay of oocytes is needed in further experiments.

Surprisingly, supplementation of IVC medium with either Good FF or miRNAs associated with Good FF supported early embryonic development. The presence of FF in the oviduct was first suggested decades ago.^35^ The protein content of the OF differs between the ipsilateral and contralateral ovulated ovaries in cows.^18^ The size and concentration of EV are similar in OF and FF cows.^10^ Moreover, miRNA profiles in the EV of OF change after ovulation.^19^ Based on these reports, miRNAs in EVs of OF are potentially derived from those in FF. Recently, we reported that miRNAs found with high frequency in OFs support embryonic development in cows.^20^ In addition, the study^20^ showed that miR‐151‐3p and miR‐425‐5p were contained in the OFs with high frequency (DDBJ Read Archive under accession number DRA011145). Here, we report for the first time that embryos take up miRNAs in EV, and supplementation of IVC medium with miR‐151‐3p and miR‐425‐5p improves the rate of development to the blastocyst stage. Furthermore, supplementation of IVC medium with FFs containing miR‐151‐3p and miR‐425‐5p (Predicted Good FF) supports embryonic development suggesting that the miRNAs associated with Good FFs support embryonic development. Interestingly, concentration of miR‐151‐3p and miR‐425‐5p significantly correlated in the FFs. In the supplementary experiment, we examined the correlation between miR‐151‐3p and miR‐148a (top miRNA, Table 3) in the FFs and found no significant correlation (Figure S2). In addition, the miRNAs were coded on different chromosomes in cows, indicating that the same transcriptional factors may regulated these miRNA expressions. Furthermore, we previously showed that miRNA profiles in GCs differ from the profiles in the spent culture medium of GCs.^21^ These results suggest that certain groups of miRNAs are selectively packaged into the EV for secretion.

We previously conducted RNA‐seq using miR‐17‐5p treated embryos or miR‐19b treated GCs. We observed that the pathways enriched by the differentially expressed genes overlapped with pathways enriched using the in silico predicted target genes of the miRNAs. Therefore, here we conducted an in silico prediction of the pathways associated with the target genes of miR‐151‐3p and miR‐425‐5p. The MAPK signaling, focal adhesion, neurotrophins, and insulin signaling pathways were affected by the treatment with miRNAs. Specifically, these pathways overlap with those reported in a previous study using target genes of miRNAs in EVs of FFs in cows,^24^ suggesting that these pathways are associated with ovarian follicular growth and oocyte maturation. However, the distinct targets of the miR‐151‐3p and miR‐425‐5p should be determined in further experiments that analyze the expressions of proteins.

Here, we show that miRNAs affect oocyte maturation and embryonic development. However, FFs contain a myriad of molecules that can affect oocyte maturation and embryonic development. To examine this, we measured the TGFB1, E_2_, and P_4_ levels in Good and Poor FFs. These measurements were made because TGFB1 affects oocyte maturation and embryonic development,^36^, ^37^ since E_2_ and P_4_ reflect the follicle condition.^38^ However, no significant differences were observed between Good FF and Poor FF (Figure S3). These results support the idea that miRNAs in EVs of FFs play significant roles in oocyte maturation and early embryonic development. However, it is still conceivable that other factors have a role in the beneficial effect of Good FF.

In conclusion, miR‐151‐3p and miR‐425‐5p found in FF determine the oocyte maturation and embryonic development and are a background for high embryo yield in cows.

## CONFLICT OF INTEREST STATEMENT

Authors declare that there are no conflicts of interest for this article. Hisataka IWATA is an Editorial Board member of Reproductive Medicine and Biology and a co‐author of this article. To minimize bias, they were excluded from all editorial decision‐making related to the acceptance of this article for publication.

## ANIMAL STUDIES AND APPROVAL BY ETHICS COMMITTEE

Bovine ovaries were routinely discarded from slaughterhouses and donated to us in this study.

This study was approved by the Ethical Committee for Animal Experiments of Tokyo University of Agriculture (2023009).

## HUMAN RIGHTS

This study does not contain any studies with human subjects.

## Supporting information


Figure S1.

Figure S2.

Figure S3.

Table S1.

## Data Availability

All datasets generated from RNA‐seq and small RNA‐seq have been registered in DDBJ. All datasets will be provided by the corresponding author upon proper request.

## References

[1] Balaban B , Urman B , Sertac A , Alatas C , Aksoy S , Mercan R . Blastocyst quality affects the success of blastocyst‐stage embryo transfer. Fertil Steril. 2000;74:282–287. 10.1016/S0015-0282(00)00645-2 10927045

[2] Lee M‐J , Lee RK‐K , Lin M‐H , Hwu Y‐M . Cleavage speed and implantation potential of early‐cleavage embryos in IVF or ICSI cycles. J Assist Reprod Genet. 2012;29:745–750. 10.1007/s10815-012-9777-z 22825967 PMC3430780

[3] Sirard M‐A , Richard F , Blondin P , Robert C . Contribution of the oocyte to embryo quality. Theriogenology. 2006;65:126–136. 10.1016/j.theriogenology.2005.09.020 16256189

[4] Somfai T , Inaba Y , Watanabe S , Geshi M , Nagai T . Follicular fluid supplementation during in vitro maturation promotes sperm penetration in bovine oocytes by enhancing cumulus expansion and increasing mitochondrial activity in oocytes. Reprod Fertil Dev. 2012;24:743–752. 10.1071/RD11251 22697124

[5] Romero‐Arredondo A , Seidel GE . Effects of follicular fluid during in vitro maturation of bovine oocytes on in vitro fertilization and early embryonic Development1. Biol Reprod. 1996;55:1012–1016. 10.1095/biolreprod55.5.1012 8902211

[6] Lopes JS , Canha‐Gouveia A , París‐Oller E , Coy P . Supplementation of bovine follicular fluid during in vitro maturation increases oocyte cumulus expansion, blastocyst developmental kinetics, and blastocyst cell number. Theriogenology. 2019;126:222–229. 10.1016/j.theriogenology.2018.12.010 30590243

[7] da Silveira JC , Andrade GM , del Collado M , Sampaio RV , Sangalli JR , Silva LA , et al. Supplementation with small‐extracellular vesicles from ovarian follicular fluid during in vitro production modulates bovine embryo development. PloS One. 2017;12:e0179451. 10.1371/journal.pone.0179451 28617821 PMC5472319

[8] Gabryś J , Kij‐Mitka B , Sawicki S , Kochan J , Nowak A , Łojko J , et al. Extracellular vesicles from follicular fluid may improve the nuclear maturation rate of in vitro matured mare oocytes. Theriogenology. 2022;188:116–124. 10.1016/j.theriogenology.2022.05.022 35689941

[9] Hung W‐T , Hong X , Christenson LK , McGinnis LK . Extracellular vesicles from bovine follicular fluid support cumulus Expansion1. Biol Reprod. 2015;93:117. 10.1095/biolreprod.115.132977 26423123 PMC4712005

[10] Asaadi A , Dolatabad NA , Atashi H , Raes A , Van Damme P , Hoelker M , et al. Extracellular vesicles from follicular and Ampullary fluid isolated by density gradient ultracentrifugation improve bovine embryo development and quality. IJMS. 2021;22:578. 10.3390/ijms22020578 33430094 PMC7826877

[11] Machtinger R , Laurent LC , Baccarelli AA . Extracellular vesicles: roles in gamete maturation, fertilization and embryo implantation. Hum Reprod Update. 2015;22:dmv055–dmv193. 10.1093/humupd/dmv055 PMC4755440 26663221

[12] Lei L , Jin S , Gonzalez G , Behringer RR , Woodruff TK . The regulatory role of Dicer in folliculogenesis in mice. Mol Cell Endocrinol. 2010;315:63–73. 10.1016/j.mce.2009.09.021 19799966 PMC2814883

[13] Kaneda M , Tang F , O'Carroll D , Lao K , Surani MA . Essential role for Argonaute2 protein in mouse oogenesis. Epigenetics Chromatin. 2009;2:9. 10.1186/1756-8935-2-9 19664249 PMC2736168

[14] Machtinger R , Rodosthenous RS , Adir M , Mansour A , Racowsky C , Baccarelli AA , et al. Extracellular microRNAs in follicular fluid and their potential association with oocyte fertilization and embryo quality: an exploratory study. J Assist Reprod Genet. 2017;34:525–533. 10.1007/s10815-017-0876-8 28188594 PMC5401697

[15] Feng R , Sang Q , Zhu Y , Fu W , Liu M , Yan X , et al. MiRNA‐320 in the human follicular fluid is associated with embryo quality in vivo and affects mouse embryonic development in vitro. Sci rep. 2015;5:8689. 10.1038/srep08689 25732513 PMC4346788

[16] Inoue Y , Munakata Y , Shinozawa A , Kawahara‐Miki R , Shirasuna K , Iwata H . Prediction of major microRNAs in follicular fluid regulating porcine oocyte development. J Assist Reprod Genet. 2020;37:2569–2579. 10.1007/s10815-020-01909-0 32780318 PMC7550524

[17] Zhan S , Paik A , Onyeabor F , Ding B , Prabhu S , Wang J . Oral bioavailability evaluation of Celastrol‐encapsulated silk fibroin nanoparticles using an optimized LC‐MS/MS method. Molecules. 2020;25:3422. 10.3390/molecules25153422 32731529 PMC7435660

[18] Mahé C , Lavigne R , Com E , Pineau C , Locatelli Y , Zlotkowska AM , et al. Spatiotemporal profiling of the bovine oviduct fluid proteome around the time of ovulation. Sci rep. 2022;12:4135. 10.1038/s41598-022-07929-3 35264682 PMC8907256

[19] Almiñana C , Tsikis G , Labas V , Uzbekov R , da Silveira JC , Bauersachs S , et al. Deciphering the oviductal extracellular vesicles content across the estrous cycle: implications for the gametes‐oviduct interactions and the environment of the potential embryo. BMC Genomics. 2018;19:622. 10.1186/s12864-018-4982-5 30134841 PMC6103977

[20] Aoki S , Inoue Y , Shinozawa A , Tanaka K , Shirasuna K , Iwata H . miR‐17‐5p in bovine oviductal fluid affects embryo development. Mol Cell Endocrinol. 2022;551:111651. 10.1016/j.mce.2022.111651 35452772

[21] Nagata S , Inoue Y , Sato T , Tanaka K , Shinozawa A , Shirasuna K , et al. Age‐associated changes in miRNA profile of bovine follicular fluid. Reproduction. 2022;164:195–206. 10.1530/REP-22-0036 35980236

[22] Ireland JJ , Murphee RL , Coulson PB . Accuracy of predicting stages of bovine estrous cycle by gross appearance of the corpus luteum. J Dairy Sci. 1980;63:155–160. 10.3168/jds.S0022-0302(80)82901-8 7372895

[23] Kim K , Mitsumizo N , Fujita K , Utsumi K . The effects of follicular fluid on in vitro maturation, oocyte fertilization and the development of bovine embryos. Theriogenology. 1996;45:787–799. 10.1016/0093-691X(96)00008-8 16727841

[24] Sohel MMH , Hoelker M , Noferesti SS , Salilew‐Wondim D , Tholen E , Looft C , et al. Exosomal and non‐Exosomal transport of extra‐cellular microRNAs in follicular fluid: implications for bovine oocyte developmental competence. PloS One. 2013;8:e78505. 10.1371/journal.pone.0078505 24223816 PMC3817212

[25] Ikeda S , Sugimoto M , Kume S . Lipofection of siRNA into bovine 8‐16‐cell stage embryos using zona removal and the well‐of‐the‐well culture system. J Reprod Develop. 2018;64:199–202. 10.1262/jrd.2017-137 PMC5902909 29353869

[26] Yang X , Wu LL , Chura LR , Liang X , Lane M , Norman RJ , et al. Exposure to lipid‐rich follicular fluid is associated with endoplasmic reticulum stress and impaired oocyte maturation in cumulus‐oocyte complexes. Fertil Steril. 2012;97:1438–1443. 10.1016/j.fertnstert.2012.02.034 22440252

[27] Hu M , Du Z , Zhou Z , Long H , Ni Q . Effects of serum and follicular fluid on the in vitro maturation of canine oocytes. Theriogenology. 2020;143:10–17. 10.1016/j.theriogenology.2019.11.040 31830685

[28] Dell'Aquila ME , Cho YS , Minoia P , Traina V , Lacalandra GM , Maritato F . Effects of follicular fluid supplementation of in‐vitro maturation medium on the fertilization and development of equine oocytes after in vitro fertilization or intracytoplasmic sperm injection. Hum Reprod. 1997;12:2766–2772. 10.1093/humrep/12.12.2766 9455850

[29] Ogawa K , Itami N , Ueda M , Kansaku K , Shirasuna K , Kuwayama T , et al. Non‐esterified fatty acid‐associated ability of follicular fluid to support porcine oocyte maturation and development. Reprod Med Biol. 2018;17:155–163. 10.1002/rmb2.12084 29692673 PMC5902458

[30] Ali A , Coenen K , Bousquet D , Sirard M‐A . Origin of bovine follicular fluid and its effect during in vitro maturation on the developmental competence of bovine oocytes. Theriogenology. 2004;62:1596–1606. 10.1016/j.theriogenology.2004.03.011 15511547

[31] Rodrigues TA , Tuna KM , Alli AA , Tribulo P , Hansen PJ , Koh J , et al. Follicular fluid exosomes act on the bovine oocyte to improve oocyte competence to support development and survival to heat shock. Reprod Fertil Dev. 2019;31:888–897. 10.1071/RD18450.0 30760387

[32] Rooda I , Hasan MM , Roos K , Viil J , Andronowska A , Smolander O‐P , et al. Cellular, extracellular and extracellular vesicular miRNA profiles of pre‐ovulatory follicles indicate signaling disturbances in polycystic ovaries. IJMS. 2020;21:9550. 10.3390/ijms21249550 33333986 PMC7765449

[33] Sørensen AE , Wissing ML , Englund ALM , Dalgaard LT . MicroRNA species in follicular fluid associating with polycystic ovary syndrome and related intermediary phenotypes. J Clin Endocrinol Metabol. 2016;101:1579–1589. 10.1210/jc.2015-3588 PMC4880172 26771704

[34] Hu J , Dong J , Zeng Z , Wu J , Tan X , Tang T , et al. Using exosomal miRNAs extracted from porcine follicular fluid to investigate their role in oocyte development. BMC Vet Res. 2020;16:485. 10.1186/s12917-020-02711-x 33317549 PMC7737261

[35] Hansen C , Srikandakumar A , Downey BR . Presence of follicular fluid in the porcine oviduct and its contribution to the acrosome reaction. Mol Reprod Dev. 1991;30:148–153. 10.1002/mrd.1080300211 1954028

[36] Hara S , Inoue Y , Aoki S , Tanaka K , Shirasuna K , Iwata H . Beneficial effect of polysaccharide gel made of xanthan gum and locust bean gum on bovine oocytes. Int J Mol Sci. 2023;24:3508. 10.3390/ijms24043508 36834915 PMC9963600

[37] Neira JA , Tainturier D , Peña MA , Martal J . Effect of the association of IGF‐I, IGF‐II, bFGF, TGF‐beta1, GM‐CSF, and LIF on the development of bovine embryos produced in vitro. Theriogenology. 2010;73:595–604. 10.1016/j.theriogenology.2009.10.015 20035987

[38] Dieleman SJ , Kruip TA , Fontijne P , de Jong WH , van der Weyden GC . Changes in oestradiol, progesterone and testosterone concentrations in follicular fluid and in the micromorphology of preovulatory bovine follicles relative to the peak of luteinizing hormone. J Endocrinol. 1983;97:31–42. 10.1677/joe.0.0970031 6682433

