# Supplementary material for: microRNAs associated with the quality of follicular fluids affect oocyte and early embryonic development
Source: Reprod Med Biol. 2024 Jan 18;23(1):e12559. doi: 10.1002/rmb2.12559 (PMC10795439; doi:10.1002/rmb2.12559)
Supplement: Supplementary file 1 — Figure S1. [file RMB2-23-e12559-s001.docx]

Supplementary Figure S1


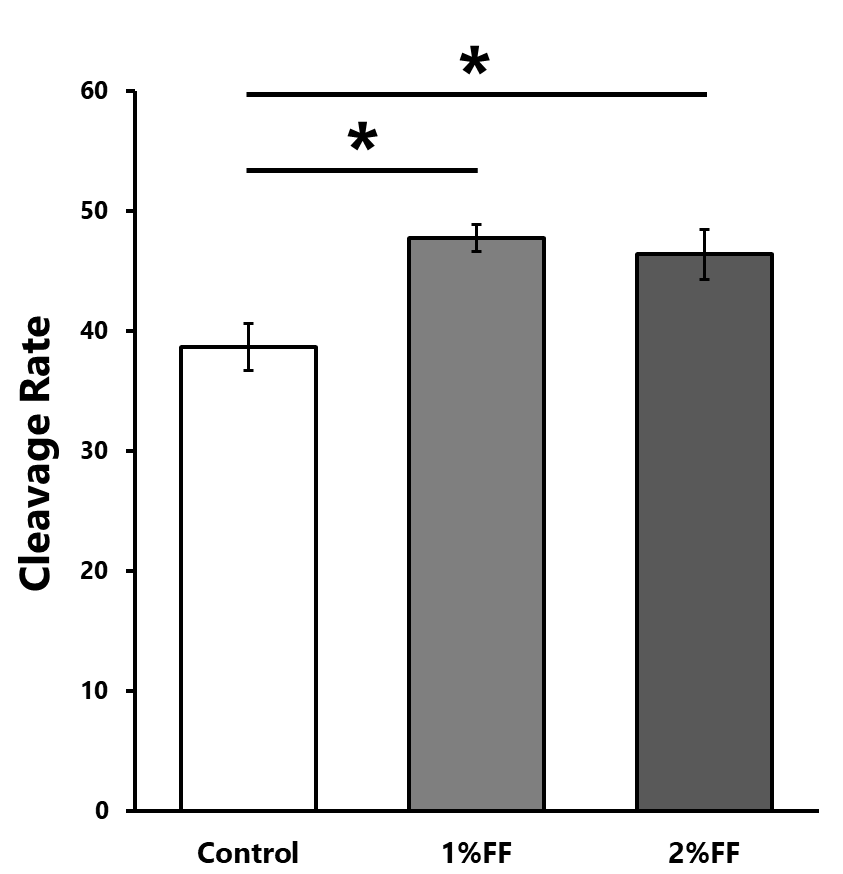


The effect of supplementation of IVC medium with 0% (Control), 1%, or 2% FF on early embryonic development. FFs were collected from 30 cows and mixed. The cleavage rate to 8-cell stage embryos were improved after supplementation of IVC medium with 1% or 2% FFs compared with that of the Control group in four trials (Control vs. 1% vs. 2%:38.6 ± 2.0% vs. 47.7 ± 1.1% vs. 46.4 ± 2.1%, respectively; *P < 0.05). Based on the results and the presumption that concentration of FFs in the oviductal fluid is low, we selected 1% FFs for subsequent experiments.
