# Supplementary material for: microRNAs associated with the quality of follicular fluids affect oocyte and early embryonic development
Source: Reprod Med Biol. 2024 Jan 18;23(1):e12559. doi: 10.1002/rmb2.12559 (PMC10795439; doi:10.1002/rmb2.12559)
Supplement: Supplementary file 2 — Figure S2. [file RMB2-23-e12559-s004.docx]

Supplementary Figure S2


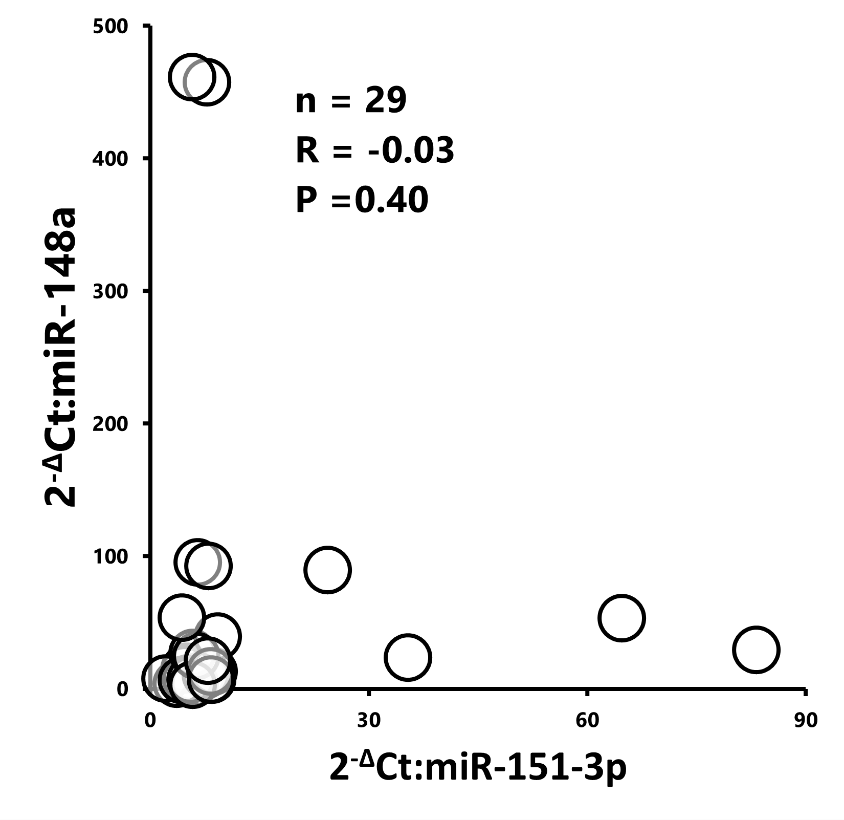


Correlation between the expression of miR-151-3p and -148a. N means number of samples, and R means correlation coefficient.
