# Supplementary material for: microRNAs associated with the quality of follicular fluids affect oocyte and early embryonic development
Source: Reprod Med Biol. 2024 Jan 18;23(1):e12559. doi: 10.1002/rmb2.12559 (PMC10795439; doi:10.1002/rmb2.12559)
Supplement: Supplementary file 3 — Figure S3. [file RMB2-23-e12559-s003.docx]

Supplementary Figure S3


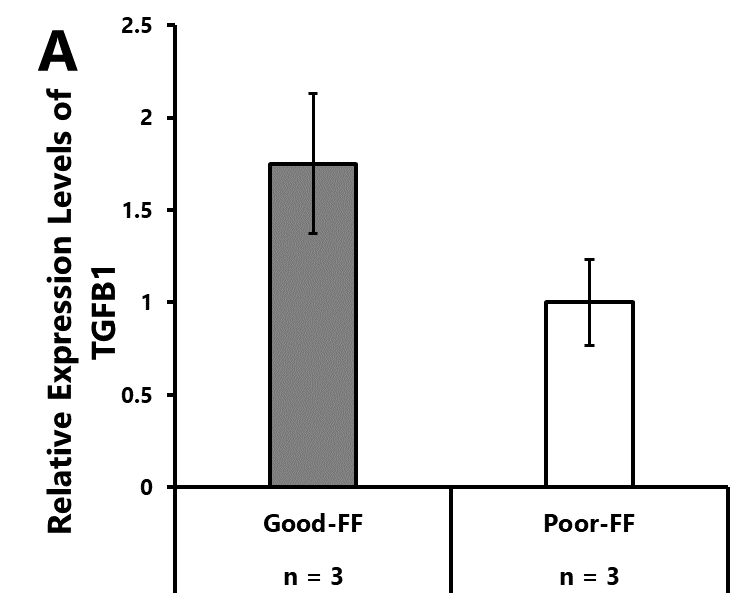

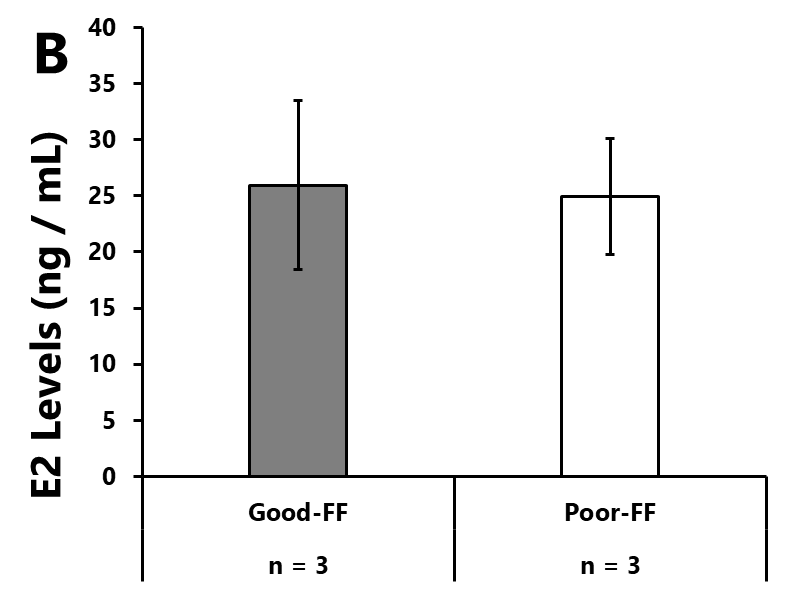

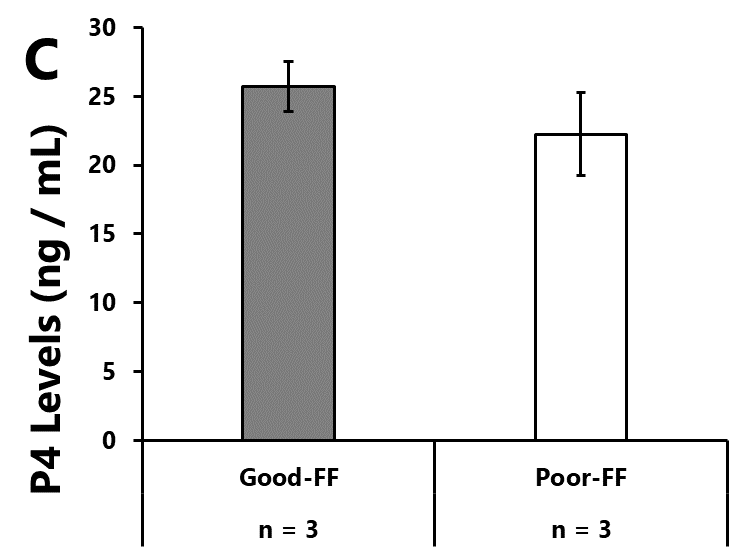


Concentration of TGFB1 (A), estradiol (E2, B), and progesterone (P4, C) in Good- and Poor- FFs. Levels of TGFB1, E2, and P4 were respectively measured using a TGFB1 ELISA Kit (Legend MaxTM, Biolegend San Diego, CA, USA), Estradiol Assay kit (R&D Systems, Minneapolis, MN, USA), and Progesterone ELISA Kit (Cayman Chemical, Ann Arbor, MI, USA) according to the manufacturers instructions. No differences were observed between the two groups (P = 0.1 for TGFB1, P = 0.9 for E2, and P = 0.5 for P4, respectively). Data are presented as mean ± SEM.
